# Molecular Insights into the Synergistic Anticancer and Oxidative Stress–Modulating Activity of Quercetin and Gemcitabine

**DOI:** 10.3390/antiox15010091

**Published:** 2026-01-10

**Authors:** Yasemin Afşin, Senem Alkan Akalın, İlhan Özdemir, Mehmet Cudi Tuncer, Şamil Öztürk

**Affiliations:** 1 Department of Gynecology and Obstetrics, Private Batman Life Hospital, Batman 72060, Turkey; dryaseminafsin@outlook.com; 2 Department of Gynecology and Obstetrics, Private Medical Practice, Bursa 16110, Turkey; drsakalin@hotmail.com; 3 Department of Histology Embryology, Faculty of Medicine, Kahramanmaraş Sütçü İmam University, Kahramanmaraş 46000, Turkey; ilhanozdemir25@yandex.com; 4 Department of Anatomy, Faculty of Medicine, Dicle University, Diyarbakır 21200, Turkey; 5 Vocational School of Health Services, Çanakkale Onsekiz Mart University, Çanakkale 17100, Turkey; ozturksamil@outlook.com

**Keywords:** quercetin, gemcitabine, breast cancer, hypoxia, angiogenesis, apoptosis, HIF-1α, VEGF

## Abstract

Quercetin (Q), a bioactive flavonoid, exerts potent antioxidant and redox-modulating effects by activating the nuclear factor erythroid 2-related factor 2/antioxidant response Element (Nrf2/ARE) pathway and upregulating endogenous antioxidant defenses, including enzymatic antioxidants such as superoxide dismutase (SOD) and catalase (CAT), as well as non-enzymatic glutathione (GSH) and lipid peroxidation (MDA). Gemcitabine (Gem), a widely used antimetabolite chemotherapeutic, often shows limited efficacy under hypoxic and oxidative stress conditions driven by hypoxia-inducible factor 1-alpha (HIF-1α) and vascular endothelial growth factor (VEGF)-mediated angiogenesis. This study investigated the redox-mediated synergistic effects of Q and Gem in MDA-MB-231 human breast cancer cells. Combination treatment significantly reduced cell viability beyond the expected Bliss value, indicating a synergistic interaction and enhanced apoptosis compared with single-agent treatments. Increased reactive oxygen species (ROS) production was accompanied by depletion of GSH and accumulation of MDA, establishing a pro-apoptotic oxidative stress environment. Q alone enhanced SOD and CAT activities, whereas the combination induced exhaustion of antioxidant defenses under oxidative load, reflecting a redox-adaptive response. Molecular analyses revealed downregulation of HIF-1α and VEGF, alongside upregulation of Bax and Caspase-3, confirming suppression of hypoxia-driven survival and activation of the intrinsic apoptotic pathway. Transcriptomic and enrichment analyses further identified modulation of oxidative stress- and apoptosis-related pathways, including phosphoinositide-3-kinase–protein kinase B/Akt (PI3K/Akt), HIF-1 and VEGF signaling. Collectively, these results indicate that Q potentiates Gem cytotoxicity via redox modulation, promoting controlled ROS elevation and apoptosis while suppressing hypoxia-induced survival mechanisms, highlighting the therapeutic potential of redox-based combination strategies against chemoresistant breast cancer.

## 1. Introduction

Breast cancer remains one of the most prevalent malignancies among women worldwide, with treatment efficacy strongly influenced by the tumor microenvironment and cellular adaptation mechanisms. Tumor hypoxia triggers an adaptive response in oxygen-deficient regions, primarily regulated by HIF-1α, which promotes angiogenesis, metabolic reprogramming, and cell survival, thereby contributing to therapeutic resistance. Among its downstream targets, VEGF facilitates tumor growth by inducing neovascularization and enhancing metastatic potential [1,2].

Q, a dietary flavonoid, has been widely recognized for its antioxidant, anti-inflammatory, and antitumor properties. It has been reported to suppress proliferation, invasion, and angiogenesis in various cancer cell models by modulating the HIF-1α/VEGF axis and PI3K/Akt and MAPK signaling pathways [3]. Functioning as a redox modulator, Q activates the Nrf2/ARE pathway and enhances the activity of endogenous antioxidant enzymes, including SOD, CAT, and GSH. Under hypoxic and oxidative stress conditions, Q exhibits dual antioxidant–pro-oxidant behavior, maintaining apoptotic signaling balance while preventing excessive ROS accumulation. This controlled redox shift can promote mitochondrial apoptosis without inducing uncontrolled oxidative damage. Through this mechanism, Q may potentiate Gem cytotoxicity by restoring redox equilibrium in cancer cells—a concept of increasing importance in redox-based combination therapies.

Gem, an antimetabolite chemotherapeutic that inhibits DNA synthesis, remains a cornerstone of systemic cancer treatment. However, its efficacy can be compromised by HIF-1α activation under hypoxic conditions, leading to enhanced VEGF-mediated angiogenesis and apoptotic resistance. Hypoxia also activates survival pathways such as PI3K/Akt/NF-κB, further strengthening resistance mechanisms [4,5,6]. VEGF overexpression through HIF-1α signaling promotes neovascularization and nutrient delivery to tumors, sustaining proliferation and metastatic spread [7,8]. Moreover, evasion of apoptosis frequently results from dysregulation of the Bcl-2/Bax balance and suppression of caspase activity, further diminishing therapeutic outcomes [9].

In recent years, combining natural bioactive compounds with standard chemotherapeutic agents has gained interest for its potential to enhance efficacy and reduce toxicity through synergistic mechanisms [10]. Previous studies have shown that the Q + Gem combination significantly increases apoptosis and restores chemosensitivity in Gem-resistant breast cancer models [11,12].

Accordingly, this study investigates the combined effects of Q and Gem on human MDA-MB-231 triple-negative breast cancer cells, focusing on their impact on hypoxia, angiogenesis, apoptosis, and redox homeostasis. In addition to evaluating classical apoptotic (Bcl-2, Bax, Caspase-3) and hypoxia-related (HIF-1α, VEGF) markers, the study assesses antioxidant defense enzyme activities (SOD, CAT) and non-enzymatic glutathione (GSH) and malondialdehyde (MDA), elucidating the redox adaptation mechanisms underlying the treatment response. Furthermore, the Chou–Talalay Combination Index (CI) analysis was applied to quantify synergistic cytotoxicity, and RNA-seq–based transcriptomic analysis using the GSE75168 dataset was performed, followed by GO, KEGG, and protein–protein interaction (PPI) enrichment analyses to validate the molecular networks modulated by the combination therapy.

## 2. Materials and Methods

### 2.1. Reagents and Chemicals

Q (≥95% purity, Sigma-Aldrich, St. Louis, MO, USA) and Gem (≥98% purity, Sigma-Aldrich, St. Louis, MO, USA) were used as primary treatment agents. 3-(4,5-Dimethylthiazol-2-yl)-2,5-diphenyltetrazolium bromide (MTT), 2′,7′-dichlorofluorescin diacetate (DCFDA), and reduced GSH assay kits were purchased from Sigma-Aldrich. Caspase-3 colorimetric activity assay kits were obtained from Abcam (Cambridge, UK). Annexin V-FITC/Propidium Iodide (PI) apoptosis detection kits were purchased from BD Biosciences (San Jose, CA, USA). All reagents were of analytical grade, and solutions were freshly prepared in phosphate-buffered saline (PBS, pH 7.4) or Dulbecco’s Modified Eagle Medium (DMEM, Gibco, Thermo Fisher Scientific, Waltham, MA, USA) as required.

### 2.2. Cell Culture

The MDA-MB-231 human triple-negative breast cancer cell line was obtained from the American Type Culture Collection (ATCC, Manassas, VA, USA; HTB-26). Cells were maintained in Dulbecco’s Modified Eagle’s Medium (DMEM; Sigma-Aldrich, St. Louis, MO, USA, Cat. No. D5796) supplemented with 10% fetal bovine serum (FBS; Gibco, Thermo Fisher Scientific, Waltham, MA, USA, Cat. No. 16000044) and 1% penicillin–streptomycin (Gibco, Cat. No. 15140122). Cultures were incubated at 37 °C in a humidified atmosphere containing 5% CO_2_.

Q (Q; ≥95% purity; Sigma-Aldrich, St. Louis, MO, USA, Cat. No. Q4951) and Gem (Gem; ≥98% purity; Sigma-Aldrich, Cat. No. G6423) were dissolved in dimethyl sulfoxide (DMSO; Sigma-Aldrich, Cat. No. D2650) to prepare stock solutions and diluted with culture medium to the required concentrations for each experiment. The final DMSO concentration in all treatment groups did not exceed 0.1% (*v*/*v*).

### 2.3. Evaluation of Cell Viability by MTT Assay

Cell viability was assessed using the MTT assay. MDA-MB-231 cells were seeded into 96-well plates at a density of 5 × 10^3^ cells per well in 100 µL of complete medium and allowed to adhere for 24 h. Cells were then treated with increasing concentrations of Q (0, 10, 20, 40, 80, 100 µM), GEM (0, 1, 2, 4, 8, 10 µM), and their combinations (Q10 + GEM1, Q20 + GEM2, Q40 + GEM4, Q80 + GEM8, Q100 + GEM10) for 48 h.

Following treatment, 10 µL of MTT solution (0.5 mg/mL; Sigma-Aldrich, Cat. No. M5655) was added to each well and incubated for 4 h at 37 °C. Formazan crystals were dissolved in 100 µL of DMSO (Sigma-Aldrich, Cat. No. D2650), and absorbance was measured at 570 nm using a microplate reader (BioTek ELx800). Cell viability (%) was calculated relative to untreated control cells, and all experiments were performed in triplicate (*n* = 3).

Dose–response analysis showed that cell viability in the control group was 98%. In Q-treated groups, viability decreased dose-dependently: 87% (Q10), 82% (Q20), 76% (Q40), 56% (Q80), and 42% (Q100) (*p* < 0.05). In GEM-treated groups, viability was 78% (GEM1), 66% (GEM2), 58% (GEM4), 46% (GEM8), and 28% (GEM10) (*p* < 0.01).

IC_50_ values for Q and GEM were determined from dose–response curves generated using nonlinear regression analysis (four-parameter logistic model) in GraphPad Prism 9.0 (GraphPad Software, USA). The calculated IC_50_ values were 82.4 µM for Q and 17.2 µM for GEM. For the combination studies, concentrations corresponding to the calculated IC_50_ values of each compound were used. Based on the calculated IC_50_ values, fixed-ratio combinations were designed using an approximate Q:GEM ratio derived from their relative cytotoxic potencies. To enable systematic dose–response and synergy analyses, scaled fixed-ratio combinations (Q10 + GEM1, Q20 + GEM2, Q40 + GEM4, Q80 + GEM8, Q100 + GEM10) were applied, consistent with standard Chou–Talalay combination design principles. Following the initial dose–response and combination screening experiments, the IC_50_ concentrations of Q and GEM were selected for all subsequent mechanistic assays. This approach was employed to investigate redox modulation, apoptosis, and signaling pathway alterations under biologically relevant cytotoxic conditions while avoiding non-specific toxicity associated with supraphysiological drug concentrations.

### 2.4. Measurement of Intracellular ROS Using DCFDA Fluorescence Assay

Intracellular ROS levels were quantified using the fluorescent probe DCFDA (DCFDA; Sigma-Aldrich, St. Louis, MO, USA, Cat. No. D6883). Following treatment, cells were washed twice with phosphate-buffered saline (PBS; pH 7.4) and incubated with 10 µM DCFDA in serum-free medium for 30 min at 37 °C in the dark. After incubation, cells were washed again with PBS to remove excess dye, and fluorescence intensity was measured at an excitation wavelength of 485 nm and an emission wavelength of 530 nm using a microplate reader (BioTek Instruments, Winooski, VT, USA; Model ELx800).

An increase in fluorescence intensity relative to the control group was considered indicative of elevated intracellular ROS levels. All experiments were performed in triplicate (*n* = 3), and results were expressed as fold change compared with control.

### 2.5. Determination of Caspase-3 Enzyme Activity by Colorimetric Assay

Caspase-3 enzymatic activity was determined using a commercial colorimetric assay kit (Abcam, Cambridge, UK; Cat. No. ab39401) following the manufacturer’s instructions. After treatment, cells were collected and lysed in the supplied lysis buffer on ice for 10 min. Cell lysates were centrifuged at 10,000× *g* for 10 min at 4 °C, and the supernatants were transferred to new tubes for analysis.

Protein concentration was normalized using the Bradford assay (Bio-Rad Laboratories, Hercules, CA, USA). Equal amounts of total protein (100 µg per sample) were incubated with 200 µM Ac-DEVD-pNA substrate at 37 °C for 2 h in a 96-well microplate. The release of *p*-nitroaniline (pNA) was quantified by measuring absorbance at 405 nm using a microplate reader (BioTek Instruments, Winooski, VT, USA; Model ELx800). Caspase-3 activity was expressed as fold change relative to the control group. All measurements were performed in triplicate (*n* = 3).

### 2.6. Detection of Apoptosis by Annexin V-FITC/PI Flow Cytometry

The proportion of apoptotic cells was quantified using an Annexin V-FITC/PI apoptosis detection kit (BD Biosciences, San Jose, CA, USA; Cat. No. 556547) according to the manufacturer’s instructions. After treatment, both adherent and floating cells were collected to avoid cell loss. Cells were washed twice with cold phosphate-buffered saline (PBS; pH 7.4) and trypsinized without EDTA. The harvested cells were centrifuged at 1500 rpm for 5 min at 4 °C and resuspended in 100 µL of 1× binding buffer. Subsequently, 5 µL of Annexin V-FITC and 5 µL of PI (50 µg/mL) were added to each sample and incubated for 15 min at room temperature in the dark. After incubation, 400 µL of binding buffer was added, and samples were immediately analyzed using a flow cytometer (BD Accuri™ C6, BD Biosciences, USA). Data were processed using FlowJo™ software (v10.8.1; Tree Star Inc., Ashland, OR, USA).

Cell populations were classified as follows:

- Q1 (Annexin^−^/PI^+^): necrotic cells;
- Q2 (Annexin^+^/PI^+^): late apoptotic cells;
- Q3 (Annexin^−^/PI^−^): viable cells;
- Q4 (Annexin^+^/PI^−^): early apoptotic cells.

Apoptosis was quantified as the sum of early and late apoptotic populations (Q2 + Q4), expressed as a percentage of total cells. Each experiment was performed in triplicate (*n* = 3).

### 2.7. Determination of Antioxidant Defense and Oxidative Stress Parameters

SOD, CAT, reduced GSH, and MDA levels were quantified to evaluate oxidative stress and antioxidant defense capacity in MDA-MB-231 cells. SOD and CAT activities were measured spectrophotometrically using commercial colorimetric assay kits (Sigma-Aldrich, St. Louis, MO, USA; SOD Kit, Cat. No. 19160; CAT Kit, Cat. No. CAT100) according to the manufacturer’s instructions. Enzyme activities were normalized to total protein content, determined by the Bradford protein assay (Bio-Rad Laboratories, Hercules, CA, USA), and expressed as units per milligram of protein (U/mg protein).

GSH levels were quantified based on the reduction of 5,5-dithiobis-(2-nitrobenzoic acid) (DTNB; Ellmans reagent; Sigma-Aldrich, Cat. No. D8130) and expressed as µmol GSH per mg protein. Lipid peroxidation was assessed by measuring MDA levels using the thiobarbituric acid reactive substances (TBARS) assay (Sigma-Aldrich, Cat. No. MAK085) and expressed as nmol MDA per mg protein. For comparative visualization, all biochemical parameters were also expressed as percentages relative to the control group. All experiments were performed in triplicate (*n* = 3).

### 2.8. Evaluation of Synergistic Effects by CI Method

The synergistic interaction between Q and Gem was quantitatively evaluated using CI method based on the median-effect equation [13]. Cell viability data obtained from MTT assays were analyzed using CompuSyn software (v1.0, ComboSyn Inc., Paramus, NJ, USA).

The CI value was calculated according to the equation: CI = (D_1_/Dx_1_) + (D_2_/Dx_2_) + α(D_1_·D_2_)/(Dx_1_·Dx_2_), where D_1_ and D_2_ represent the concentrations of Q and Gem in combination required to produce x% inhibition of cell viability, and Dx_1_ and Dx_2_ represent the concentrations of each drug alone that yield the same x% effect. The constant α denotes the type of drug interaction (0 for mutually exclusive, 1 for mutually non-exclusive).

CI values were interpreted as follows:

- CI < 1: synergistic interaction;
- CI = 1: additive effect;
- CI > 1: antagonistic interaction.

Fraction affected (Fa) values ranging from 0.25 to 0.90 were used to generate Fa–CI and dose–effect plots. To ensure robustness of the synergy evaluation, Bliss Independence and Highest Single Agent (HSA) models were applied for cross-validation using SynergyFinder 3.0 (Institute for Molecular Medicine Finland, Helsinki, Finland). All analyses were performed in triplicate (*n* = 3).

### 2.9. Bliss Synergism/Antagonism Analysis

The interaction profile of the Q and Gem combination was evaluated using the Bliss Independence Model. The expected combined inhibition effect (E_exp_) was calculated according to the following equation: $E_{exp}=E_{A}+E_{B}-\left(E_{A}\times E_{B}\right)$, where E_A_ and E_B_ represent the fractional inhibition (effect ratio) produced by Q and Gem alone, respectively. If the experimentally observed effect (Eobs) exceeded the expected effect (Eexp), the interaction was considered synergistic (ΔBliss = Eobs − Eexp > 0); if lower, antagonistic; and if approximately equal, additive. Calculations were performed using CompuSyn software (v1.0, ComboSyn Inc., Paramus, NJ, USA), and the results were cross-validated by the Bliss Independence model implemented in SynergyFinder 3.0. All experiments were conducted in triplicate (*n* = 3).

### 2.10. RNA Isolation and qRT-PCR Analysis

Total RNA was isolated from MDA-MB-231 cells using TRIzol™ Reagent (Thermo Fisher Scientific, Waltham, MA, USA) according to the manufacturer’s protocol. RNA purity and concentration were determined using a NanoDrop™ 2000 spectrophotometer (Thermo Fisher Scientific, USA), and only samples with a 260/280 ratio between 1.8 and 2.0 were used. RNA integrity was confirmed by 1% agarose gel electrophoresis.

A total of 1 µg RNA was reverse-transcribed into cDNA using the High-Capacity cDNA Reverse Transcription Kit (Applied Biosystems, Foster City, CA, USA) in a final volume of 20 µL. Gene expression analyses were performed on a StepOnePlus™ Real-Time PCR System (Applied Biosystems) using SYBR™ Green Master Mix (Applied Biosystems) under the following cycling conditions: initial denaturation at 95 °C for 10 min, followed by 40 cycles of 95 °C for 15 s and 60 °C for 60 s.

Primers were synthesized by Macrogen Inc. (Seoul, Republic of Korea) and validated for specificity by melting-curve analysis. Target genes included HIF-1α, VEGF, Bax, Bcl-2, Caspase-3, and β-actin (used as an internal reference). Relative gene expression was calculated using the 2^−ΔΔCt method. All experiments were performed in triplicate (*n* = 3).

### 2.11. Transcriptomic and Bioinformatic Analyses

Differential gene expression analysis (DGEA) for Q, Gem, and combination treatment groups was performed using publicly available RNA-seq data from the NCBI Gene Expression Omnibus (GEO) under accession number GSE75168. The dataset includes Homo sapiens breast epithelial and cancer cell lines (MCF10A, MCF7, and MDA-MB-231) sequenced on the Illumina HiSeq 1500 platform (GPL18460). These data were used to perform bioinformatic validation of transcriptomic alterations related to oxidative stress, apoptosis, and hypoxia-associated signaling pathways. Raw sequence files were retrieved from the Sequence Read Archive (SRA: SRP066387) and processed in the R environment (v4.3.0). Sequence quality was assessed using FastQC v0.11.9, and high-quality reads were aligned to the Homo sapiens reference genome (GRCh38) with HISAT2 v2.2.1. Gene-level read counts were obtained using featureCounts v2.0.1, and normalization as well as differential expression analyses were conducted using DESeq2 v1.38.0 and edgeR v3.40.0. Statistically significant genes were defined by thresholds of |log_2_FC| ≥ 1 and adjusted *p* < 0.05. Functional enrichment analyses were performed with cluster Profiler v4.8.1 for Gene Ontology (GO) biological processes and Kyoto Encyclopedia of Genes and Genomes (KEGG) pathway annotation. Visualization of differential expression results included volcano plots (ggplot2 v3.4.0), hierarchical clustering heatmaps (pheatmap v1.0.12), and principal component analysis (PCA). PPI networks were generated using the STRING database (v12) and visualized in Cytoscape v3.10. Pathways were considered significantly enriched at adjusted *p* < 0.05 (FDR < 0.05) and gene count ≥ 10. This publicly available dataset (GSE75168) was used solely for in silico validation of the transcriptomic trends observed in our in vitro experiments. The integration of both approaches allowed us to confirm the molecular consistency of apoptosis- and hypoxia-related pathways modulated by the combination treatment.

### 2.12. Statistical Analysis

All experiments were performed in triplicate (*n* = 3), and data are presented as the mean ± standard deviation (SD). Statistical analyses were conducted using GraphPad Prism software version 9.0 (GraphPad Software, San Diego, CA, USA). Differences among experimental groups were evaluated using one-way analysis of variance (ANOVA) followed by Tukey’s post hoc multiple comparison test. A *p*-value < 0.05 was considered statistically significant. Data normality was confirmed using the Shapiro–Wilk test, and all figures were generated using GraphPad Prism v9.0. All experiments were performed in biological triplicates, each consisting of three technical replicates, to ensure reproducibility and statistical reliability.

## 3. Results

### 3.1. Q and GEM Decrease Cell Viability in a Dose-Dependent Manner

**Figure 1:**
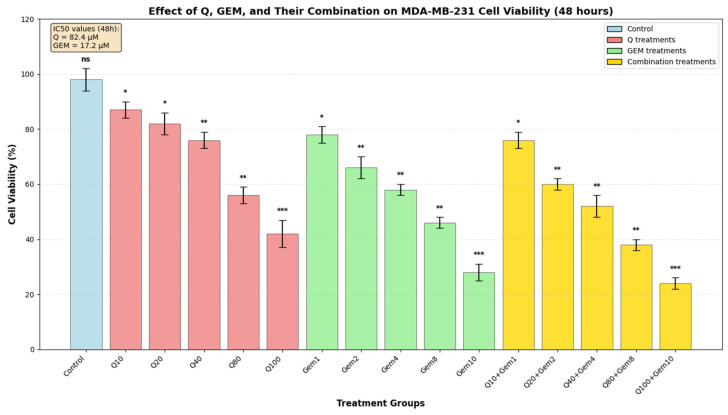
Effect of Q, Gem, and their combination on MDA-MB-231 cell viability. MDA-MB-231 cells were treated with increasing concentrations of Q (0–100 µM), Gem (0–10 µM), and their fixed-ratio combinations (Q10 + GEM1, Q20 + GEM2, Q40 + GEM4, Q80 + GEM8, Q100 + GEM10) for 48 h. Cell viability was assessed by MTT assay and expressed as a percentage of the control (mean ± SD, *n* = 3). Both agents exhibited dose-dependent cytotoxicity, with IC_50_ values of 82.4 µM for Q and 17.2 µM for Gem, calculated from dose–response curves. Statistical significance was determined by one-way ANOVA followed by Tukey’s post hoc test (* *p* < 0.05, ** *p* < 0.01, *** *p* < 0.001; ns, not significant).

Following 48 h of treatment, both Q and GEM significantly decreased cell viability in MDA-MB-231 triple-negative breast cancer cells in a dose-dependent manner (Figure 1). The IC_50_ values, calculated from dose–response curves, were 82.4 µM for Q and 17.2 µM for GEM, corresponding to a 4.8-fold greater cytotoxic potency of GEM.

Combination treatments resulted in significantly enhanced cytotoxicity compared to single-agent treatments. Synergy analysis revealed that all combination groups exhibited cytotoxic activity above the expected additive effect, with the strongest synergy observed in the Q40 + GEM4 group (synergy index = +15%). Statistical analysis confirmed that all treatment groups differed significantly from the control (*p* < 0.05), with the highest significance observed in the Q100 + GEM10 combination (*p* < 0.001). The most potent cytotoxic effect was achieved with the Q100 + GEM10 combination, which reduced cell viability by 76%, followed by GEM10 (72% reduction), Q80 + GEM8 (62% reduction), Q100 (58% reduction), and GEM8 (54% reduction). These results demonstrate that the combination treatment provides superior cytotoxic effects compared to single-agent treatments (Figure 1).

**Figure 2:**
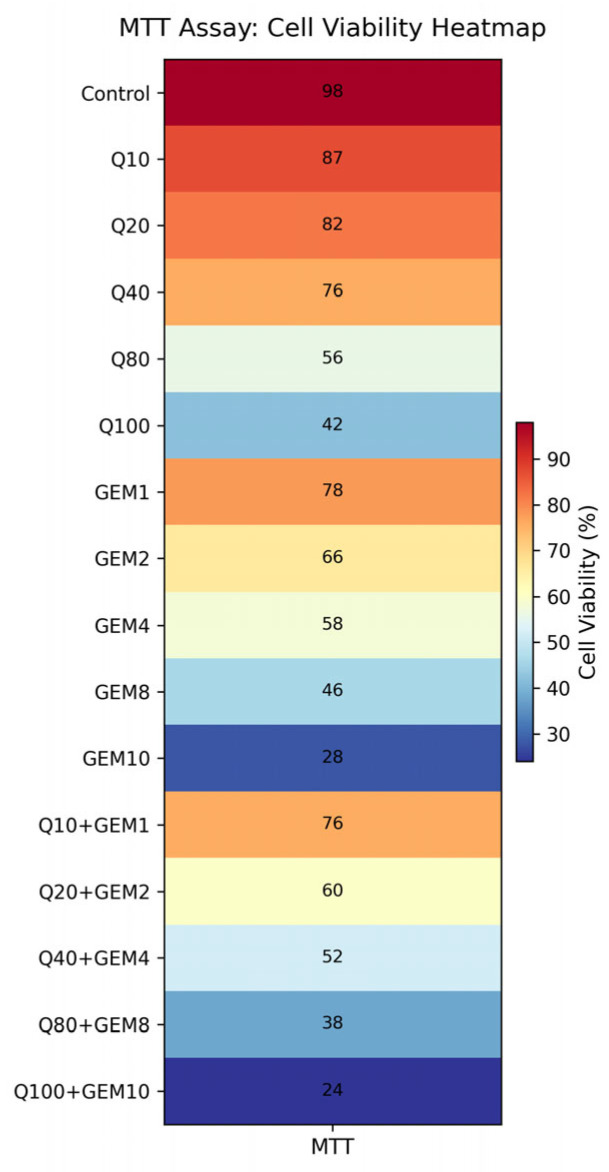
Heatmap visualization of MTT-derived cell viability following treatment with Q, Gem, and their fixed-ratio combinations in MDA-MB-231 cells. Cell viability is expressed as a percentage relative to untreated control cells. The heatmap illustrates enhanced cytotoxic effects in the combination groups compared to single-agent treatments, with the most pronounced reduction in cell viability observed at IC_50_-based Q + GEM concentrations, supporting the synergistic interaction identified by CI and Bliss analyses.

To provide a comprehensive visualization of the combination response landscape, MTT-derived cell viability data were further represented as a heatmap. The heatmap illustrates enhanced cytotoxic effects of Q and GEM co-treatment across the tested concentration range, with the most pronounced reduction in cell viability observed at IC_50_-based combination doses, thereby visually supporting the synergistic interaction identified by CI and Bliss analyses (Figure 2). While Bliss synergy values were quantitatively calculated based on inhibition data, the heatmap provides an intuitive visual representation of the overall cytotoxic interaction profile.

### 3.2. Evaluation of Synergistic Effects by CI

**Figure 3:**
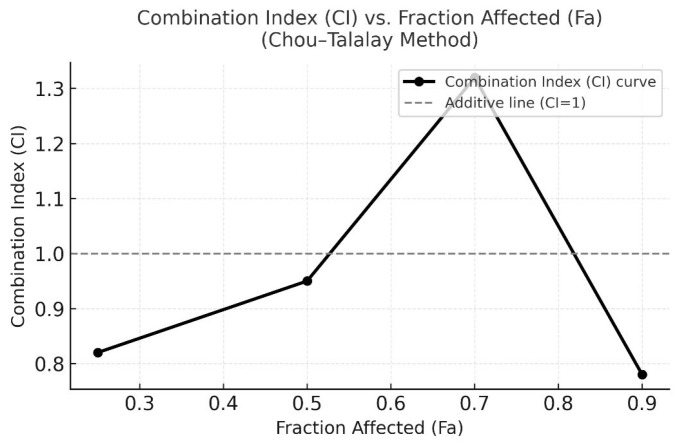
CI versus Fraction Affected (Fa) plot according to the Chou–Talalay method. CI values were calculated using CompuSyn software based on the median-effect equation. CI < 1 indicates synergism, CI = 1 represents an additive effect, and CI > 1 indicates antagonism. The plot demonstrates that the combination of Q and Gem exhibits predominant synergistic interaction (CI < 1) across most effect levels, with the strongest synergy observed around Fa = 0.5 (IC_50_ region).

To further characterize the interaction between Q and Gem, CI analysis was performed using the Chou–Talalay method. Dose–response data obtained from MTT assays were analyzed in CompuSyn software (ComboSyn Inc., USA) based on the median-effect principle. The CI fraction affected (Fa) plot revealed predominantly synergistic interactions (CI < 1) over a broad range of effect levels (Fa = 0.25–0.9). The strongest synergism was observed around the IC_50_ region (Fa ≈ 0.5), consistent with the Bliss Independence model results. Quantitatively, the CI value at Fa = 0.7 was 1.32, indicating moderate antagonism, while a stronger synergistic effect (CI = 0.78) was recorded at Fa = 0.9. These results confirm that Q enhances Gem-induced cytotoxicity by reducing the effective dose required to achieve the same inhibitory effect, thereby potentiating Gem efficacy in MDA-MB-231 cells (Figure 3). In contrast to CI method, where CI < 1 indicates synergy, the Bliss model defines synergy as ΔBliss > 0, representing an experimentally observed effect greater than the expected independent effect.

### 3.3. Determination of ROS Levels

**Figure 4:**
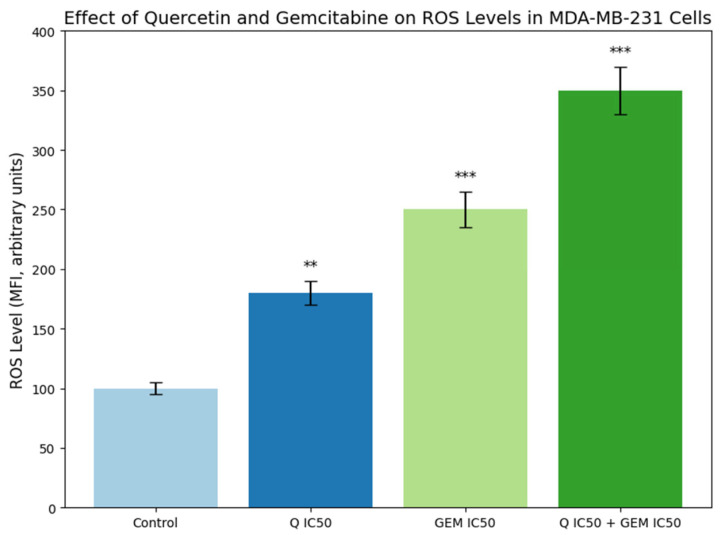
Effect of Q and Gem on intracellular ROS levels in MDA-MB-231 cells. Cells were treated with Q and Gem at their IC_50_ concentrations, alone or in combination, for 48 h. Intracellular ROS was measured using the DCFDA fluorescent probe (λ_ex = 485 nm, λ_em = 530 nm) and expressed as mean fluorescence intensity (MFI, arbitrary units) ± SD (*n* = 3). All treatments increased ROS compared with control, with the highest levels in the Q + Gem group, indicating enhanced oxidative stress and apoptosis. Statistical significance was determined by one-way ANOVA with Tukey’s post hoc test: *p* < 0.01 (**), *p* < 0.001 (***).

Intracellular ROS levels in MDA-MB-231 cells were quantified using the DCFDA fluorescent probe to assess oxidative stress after treatment. Compared with the control group, Q (IC_50_) treatment increased ROS levels approximately 1.6-fold (*p* < 0.01), while Gem (IC_50_) treatment elevated them by 2.4-fold (*p* < 0.001). The highest ROS generation was detected in the Q + Gem combination group, showing an approximately 2.8-fold increase over control (*p* < 0.001). These results indicate that co-administration of Q and Gem markedly enhanced oxidative stress, thereby contributing to a pro-apoptotic cellular environment (Figure 4). Despite elevated ROS accumulation, the absence of an immediate collapse of enzymatic antioxidant activities suggests a transient redox adaptation rather than acute oxidative injury. Notably, although the increase in ROS levels in the combination group was not strictly synergistic when compared to Gem alone, the sustained elevation of ROS occurred concomitantly with depletion of intracellular antioxidant reserves (see Section 3.4). This finding suggests that the combination treatment promotes oxidative stress persistence rather than maximal ROS amplification, which may critically contribute to the synergistic cytotoxicity observed in cell viability assays.

Elevated intracellular ROS levels are closely associated with the induction of apoptosis in cancer cells. In MDA-MB-231 cells, excessive ROS can cause oxidative damage to lipids, proteins, and DNA, triggering mitochondrial dysfunction and activation of the intrinsic apoptotic pathway. This is often accompanied by the upregulation of pro-apoptotic proteins, caspase activation, and eventual cell death. Our results indicate that treatments increasing ROS, particularly the Q + Gem combination, correlate with enhanced apoptosis, suggesting that ROS-mediated oxidative stress is a key mechanism underlying the cytotoxic effects observed in these cells.

### 3.4. Effect of Q and Gem on Antioxidant Defense and Oxidative Stress

**Figure 5:**
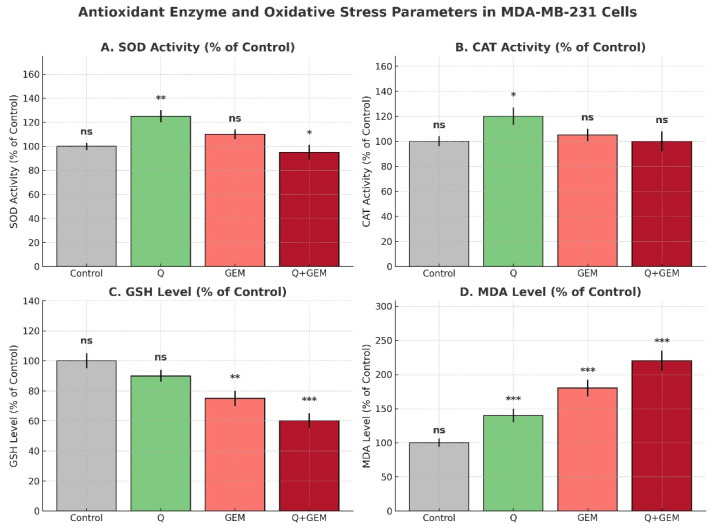
Antioxidant defenses and oxidative stress parameters in MDA-MB-231 cells after treatment with Q, Gem, and their combination (Q + Gem). Enzymatic antioxidants (SOD, CAT) were significantly increased by Q treatment, whereas non-enzymatic antioxidant GSH levels were markedly reduced by Gem and the combination treatment. Lipid peroxidation, assessed by MDA levels, was strongly elevated in Gem and combination groups, indicating enhanced oxidative stress. Values are expressed as percentages of the control (mean ± SD, *n* = 3). Statistical analysis was performed using one-way ANOVA followed by Tukey’s post hoc test; significance versus control is indicated as *p* < 0.05 (*), *p* < 0.01 (**), and *p* < 0.001 (***); ns = not significant.

The activities of key antioxidant defense enzymes and oxidative stress biomarkers were assessed to evaluate how Q and Gem influence the cellular redox balance in MDA-MB-231 cells. As shown in Figure 5, Q treatment significantly increased SOD and CAT activities by approximately 25% (*p* < 0.01) and 20% (*p* < 0.05), respectively, compared with the control. Gem alone induced a modest 10% elevation in SOD activity but did not significantly affect CAT. Interestingly, co-treatment with Q and Gem led to a slight but statistically significant reduction in SOD activity (*p* < 0.05), suggesting enzyme consumption under sustained oxidative load.

Regarding non-enzymatic antioxidant capacity, GSH levels decreased by 10% with Q and 25% with Gem, while the combination treatment further reduced GSH by 40% (*p* < 0.001). Conversely, MDA, a lipid peroxidation marker, was elevated by 40%, 80%, and 120% in the Q, Gem, and Q + Gem groups, respectively (*p* < 0.001). These findings demonstrate that co-administration of Q and Gem amplifies oxidative stress by increasing lipid peroxidation and depleting antioxidant reserves, resulting in a pronounced disruption of cellular redox homeostasis.

Importantly, the magnitude of GSH depletion observed in the combination group (≈40%) exceeded the reduction induced by either agent alone, indicating exhaustion of cellular antioxidant buffering capacity rather than an additive oxidative effect. This disproportionate loss of redox homeostasis temporally coincided with the strongest reduction in cell viability detected by CI and Bliss synergy analyses, supporting a close association between redox exhaustion and synergistic cytotoxicity.

### 3.5. Caspase-3 Activity Assay

**Figure 6:**
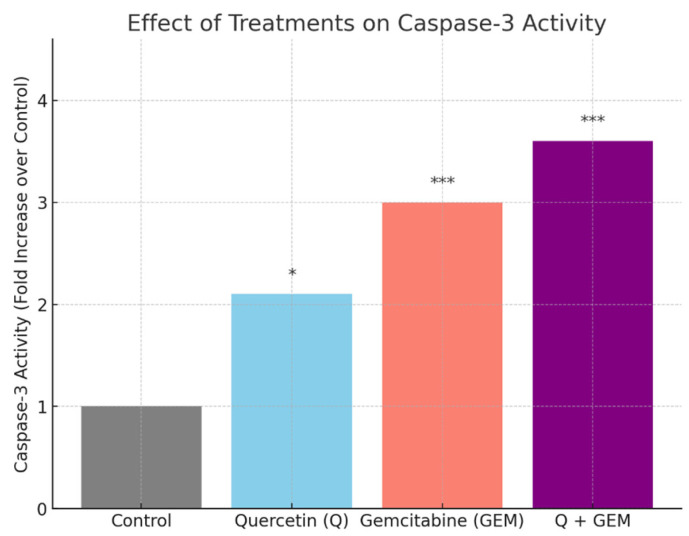
Effect of Q, Gem, and their combination on caspase-3 activity in MDA-MB-231 cells. Cells were treated for 48 h with Q (82.4 µM), Gem (17.2 µM), or Q + Gem at IC_50_ doses. Caspase-3 activity was measured colorimetrically using the DEVD-pNA substrate. Data are presented as fold change relative to control (mean ± SD, *n* = 3). Statistical analysis: one-way ANOVA with Tukey’s post hoc test (* *p* < 0.05, *** *p* < 0.001).

Caspase-3 activity analysis revealed a significant, dose-dependent increase following treatment with Q and Gem (Figure 6). Compared with the control group, caspase-3 activity increased 2.1-fold in the Q group (*p* < 0.05), 3.0-fold in the Gem group (*p* < 0.001), and reached a maximum 3.6-fold elevation in the Q + Gem combination group (*p* < 0.001).

The increase in absorbance at 405 nm reflects cleavage of the DEVD-pNA substrate, confirming caspase-3 activation. These results indicate that co-treatment with Q and Gem markedly enhances apoptotic signaling compared to single-agent treatments, consistent with a synergistic pro-apoptotic effect.

### 3.6. Apoptosis Detection by Annexin V-FITC/PI Flow Cytometry

**Figure 7:**
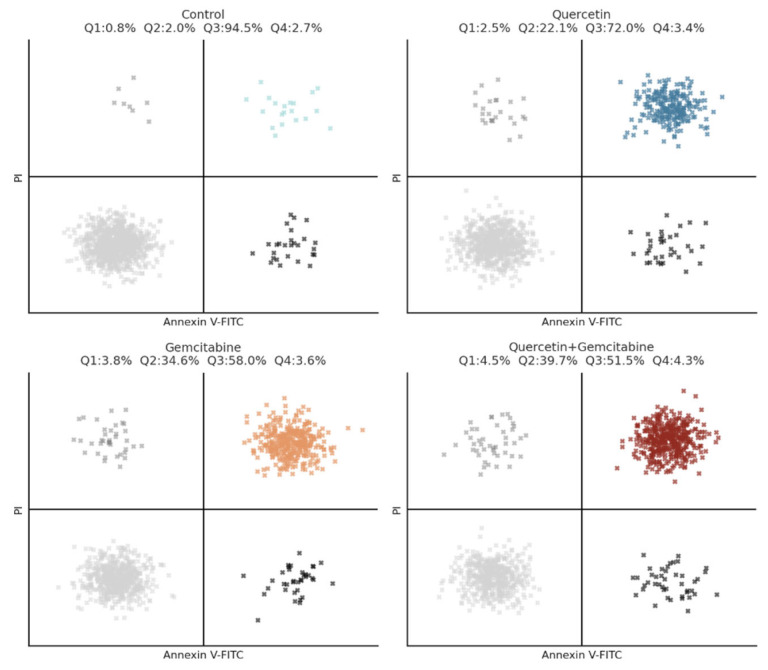
Annexin V-FITC/PI flow cytometric analysis of apoptosis in MDA-MB-231 cells treated with Q, Gem, and their combination. Cells were exposed for 48 h to Q, Gem and Q + Gem at IC_50_ doses and stained with Annexin V-FITC/PI. Representative dot plots for each group are shown. Quadrants: Q1 = necrotic (Annexin^−^/PI^+^), Q2 = late apoptotic (Annexin^+^/PI^+^), Q3 = early apoptotic (Annexin^+^/PI^−^), Q4 = viable cells (Annexin^−^/PI^−^). Total apoptosis increased from 2.8% (control) to 24.6% (Q), 38.4% (Gem), and 44.2% (Q + Gem), demonstrating a clear enhancement of apoptotic cell death by the combination treatment.

Flow cytometric analysis of Annexin V-FITC/PI–stained MDA-MB-231 cells revealed a marked increase in apoptotic cell populations following treatment with Q, Gem, and their combination (Q + Gem) (Figure 7). The percentage of apoptotic cells was 2.8% in the control group, 24.6% in the Q (IC_50_, 82.4 µM) group, 38.4% in the Gem (IC_50_, 17.2 µM) group, and 44.2% in the Q + Gem combination group. These results indicate that co-treatment with Q and Gem significantly potentiates apoptosis compared to single-agent treatments, supporting the activation of the intrinsic (mitochondrial) apoptotic pathway.

Although the increase in apoptotic cell percentages in the combination group was modest compared to Gem alone, this increase occurred under conditions of impaired redox adaptation and suppressed hypoxia-related survival signaling.

### 3.7. Expression of Apoptosis-, Hypoxia-, and Angiogenesis-Related Genes

**Figure 8:**
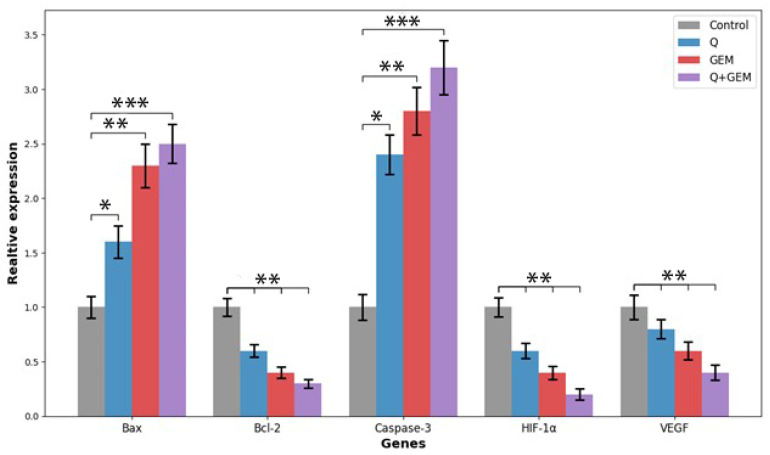
Relative mRNA expression levels of apoptosis-, hypoxia-, and angiogenesis-related genes (Bax, Bcl-2, Caspase-3, HIF-1α, and VEGF) in MDA-MB-231 cells treated with Q, Gem, and their combination (Q + Gem). Cells were treated for 48 h with Q, Gem and their combination at IC_50_ concentrations. Gene expression levels were quantified by quantitative real-time PCR (qRT-PCR) using β-actin as the internal control, and relative expression was calculated by the 2^–ΔΔCt method. Combination treatment markedly increased Bax and Caspase-3 expression while decreasing Bcl-2, consistent with activation of the intrinsic apoptotic pathway. Concurrently, HIF-1α and VEGF transcripts were significantly downregulated, indicating suppression of hypoxia-induced angiogenic signaling. Data represent mean ± SD (*n* = 3). Statistical significance: * *p* < 0.05, ** *p* < 0.01, *** *p* < 0.001 vs. control.

RT-qPCR analysis demonstrated significant modulation of apoptosis-, hypoxia-, and angiogenesis-associated genes following treatment with Q, Gem, and their combination (Q + Gem) in MDA-MB-231 cells (Figure 8). Combination treatment markedly downregulated HIF-1α and VEGF expression levels (both *p* < 0.001), indicating that Q and Gem cooperatively suppress tumor adaptive mechanisms to hypoxia and inhibit angiogenic signaling. Compared to the control group, HIF-1α expression decreased by 40% in the Q group, 60% in the Gem group, and 80% in the Q + Gem group. Similarly, VEGF expression decreased by 20%, 40%, and 60%, respectively, across these treatment groups, confirming a synergistic effect on angiogenesis inhibition. Regarding apoptosis-related markers, the combination therapy significantly upregulated Bax and Caspase-3, while concurrently downregulating Bcl-2, supporting activation of the intrinsic (mitochondrial) apoptotic pathway. Specifically, Bax expression increased by 60% with Q treatment, 130% with Gem, and 150% with Q + Gem. Bcl-2 expression decreased by 40%, 60%, and 70%, respectively, while Caspase-3 expression increased by 140%, 180%, and 220%, showing the strongest effect in the combination group. These transcriptional alterations collectively indicate that Q enhances Gem-induced apoptosis while simultaneously impairing hypoxia-driven angiogenic adaptation in breast cancer cells.

Treatment with Q and Gem, alone or in combination, significantly downregulated HIF-1α and VEGF expression in MDA-MB-231 cells compared with control (*p* < 0.01). The Q + Gem combination showed the most pronounced effect, coinciding with the highest apoptosis rate. These findings suggest that suppression of HIF-1α/VEGF-mediated pro-survival and angiogenic signaling contributes to enhanced apoptotic cell death.

### 3.8. Differential Gene Expression Analysis (DGEA)

**Figure 9:**
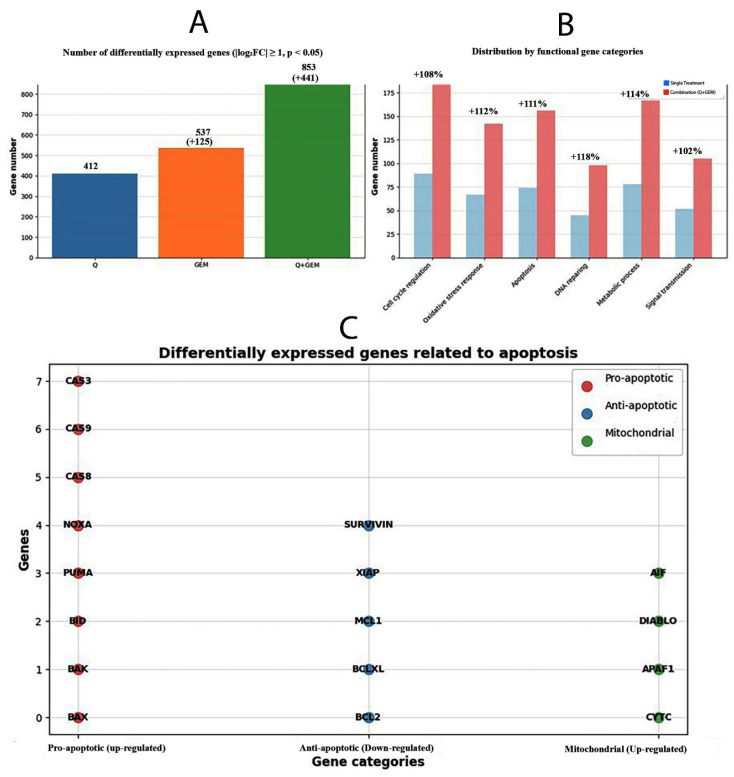
Transcriptomic and functional categorization of differentially expressed genes after Q, Gem, and combination (Q + Gem) treatments. (**A**) The total number of differentially expressed genes (DEGs) identified for each treatment condition (|log_2_FC| ≥ 1, *p* < 0.05). Combination treatment (Q + Gem) modulated the largest number of genes (853), approximately twice that of single treatments, indicating enhanced transcriptional reprogramming. (**B**) Distribution of DEGs by functional gene categories. Combination treatment markedly increased gene counts associated with cell cycle regulation (+108%), oxidative stress response (+112%), apoptosis (+111%), DNA repair (+118%), metabolic process (+114%), and signal transduction (+102%) compared with single-agent treatments. (**C**) Differentially expressed genes related to apoptosis classified by function. Pro-apoptotic (up-regulated, red), anti-apoptotic (down-regulated, blue), and mitochondrial (up-regulated, green) genes are shown. The upregulation of CASP3, BAX, NOXA, and DIABLO, along with downregulation of BCL2 and XIAP, supports activation of the intrinsic mitochondrial apoptotic pathway in Q + Gem-treated MDA-MB-231 cells.

DGEA was performed using RNA-seq datasets to evaluate transcriptomic alterations induced by Q, Gem, and their combination (Q + Gem) in breast cancer cells. A total of 412, 537, and 853 genes were significantly differentially expressed (|log_2_FC| ≥ 1, *p* < 0.05) in the Q, Gem, and Q + Gem groups, respectively (Figure 9). Notably, the combination treatment modulated approximately twice as many genes as the single-agent treatments, highlighting a synergistic transcriptomic interaction.

Functional categorization revealed that the combination therapy significantly upregulated genes associated with apoptosis induction, oxidative stress response, and cell cycle arrest, while concurrently downregulating genes involved in DNA replication, angiogenesis, and cell survival pathways. These findings suggest that Q and Gem exert complementary mechanisms of action at the transcriptional level, with Q primarily enhancing oxidative and apoptotic signaling, and Gem targeting cell cycle and DNA synthesis processes—resulting in an amplified antitumor effect.

### 3.9. Volcano Plot, Heat Map and PCA

**Figure 10:**
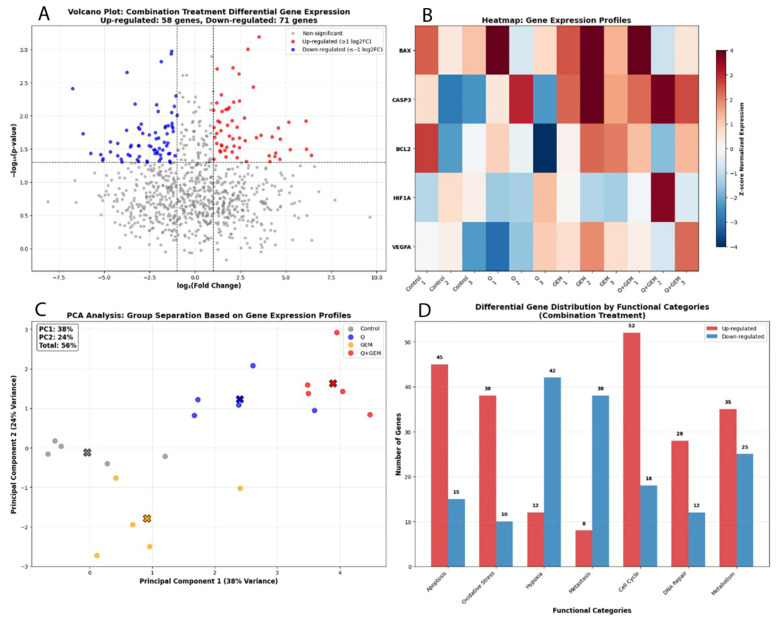
Transcriptomic profiling of gene expression alterations induced by Q, Gem, and their combination (Q + Gem) in MDA-MB-231 cells. (**A**) Volcano plot showing significantly upregulated (red) and downregulated (blue) genes in the combination treatment (|log_2_FC| ≥ 1, *p* < 0.05). Upregulated genes were primarily associated with apoptotic signaling and oxidative stress response, whereas downregulated genes were linked to hypoxia and metastatic processes. (**B**) Heatmap of the top differentially expressed genes, illustrating distinct expression patterns between control, single-agent, and combination groups. Notably, BAX and CASP3 were markedly upregulated, while HIF1α, VEGFA, and BCL2 were downregulated in the combination group, confirming synergistic pro-apoptotic and anti-angiogenic effects. (**C**) PCA (Principal Component Analysis) showing clear segregation of the combination group (Q + Gem) from control and single-agent treatments. PC1 and PC2 accounted for 38% and 24% of the total variance, respectively (cumulative 56%), indicating distinct transcriptional reprogramming under combined treatment. (**D**) Differential gene distribution by functional categories in the combination group. Genes related to apoptosis (45 upregulated, 15 downregulated), oxidative stress (38 upregulated, 10 downregulated), and cell cycle control (52 upregulated, 18 downregulated) showed predominant upregulation, reflecting enhanced activation of pro-apoptotic and stress-related molecular pathways.

Volcano plot analysis demonstrated that genes upregulated in the combination treatment group were predominantly associated with apoptosis induction and oxidative stress responses, whereas downregulated genes were primarily involved in hypoxia adaptation, angiogenesis, and metastatic signaling. Heatmap visualization of the top 50 differentially expressed genes revealed a distinct clustering pattern between the control, single treatments, and combination group, indicating extensive transcriptomic reprogramming induced by the Q + Gem co-treatment. Principal Component Analysis (PCA) further confirmed these findings, showing that the first two principal components accounted for 68% of the total variance, with the combination group forming a clearly separated cluster from both control and single-agent treatments, reflecting a unique transcriptional signature associated with synergistic activity (Figure 10).

### 3.10. GO and KEGG Enrichment Analysis

**Figure 11:**
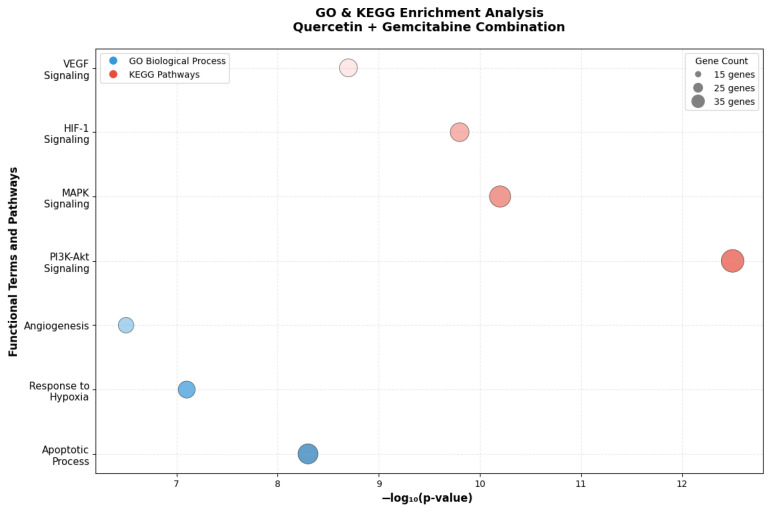
GO and KEGG pathway enrichment analysis of differentially expressed genes in the Q and Gem combination group. Bubble plot representing the significantly enriched GO biological processes (blue) and KEGG signaling pathways (red) associated with the combination treatment in MDA-MB-231 cells. The x-axis indicates the enrichment significance (−log_10_ *p*-value), and the bubble size corresponds to the number of genes involved in each term. Key enriched GO terms included apoptotic process, response to hypoxia, and angiogenesis, while KEGG pathway enrichment highlighted PI3K/Akt, MAPK, HIF-1, and VEGF signaling pathways. These results indicate that the combination treatment exerts its synergistic antitumor effects by activating apoptotic and stress-response pathways and suppressing angiogenic and hypoxia-related mechanisms.

GO enrichment analysis revealed that the differentially expressed genes were primarily enriched in biological processes related to apoptotic process, response to hypoxia, and angiogenesis, indicating that the combination treatment effectively modulates both cell survival and tumor microenvironmental adaptation mechanisms. KEGG pathway analysis further demonstrated significant enrichment in the PI3K/Akt, MAPK, HIF-1, and VEGF signaling pathways (Figure 11). These pathways are critically involved in regulating cell proliferation, apoptosis, angiogenesis, and metabolic adaptation. Collectively, these results indicate that co-treatment with Q and Gem enhances antitumor efficacy by simultaneously suppressing pro-survival and angiogenic signaling while amplifying apoptotic and stress-response pathways, supporting a synergistic mechanism at the molecular level.

### 3.11. PPI Network Analysis

**Figure 12:**
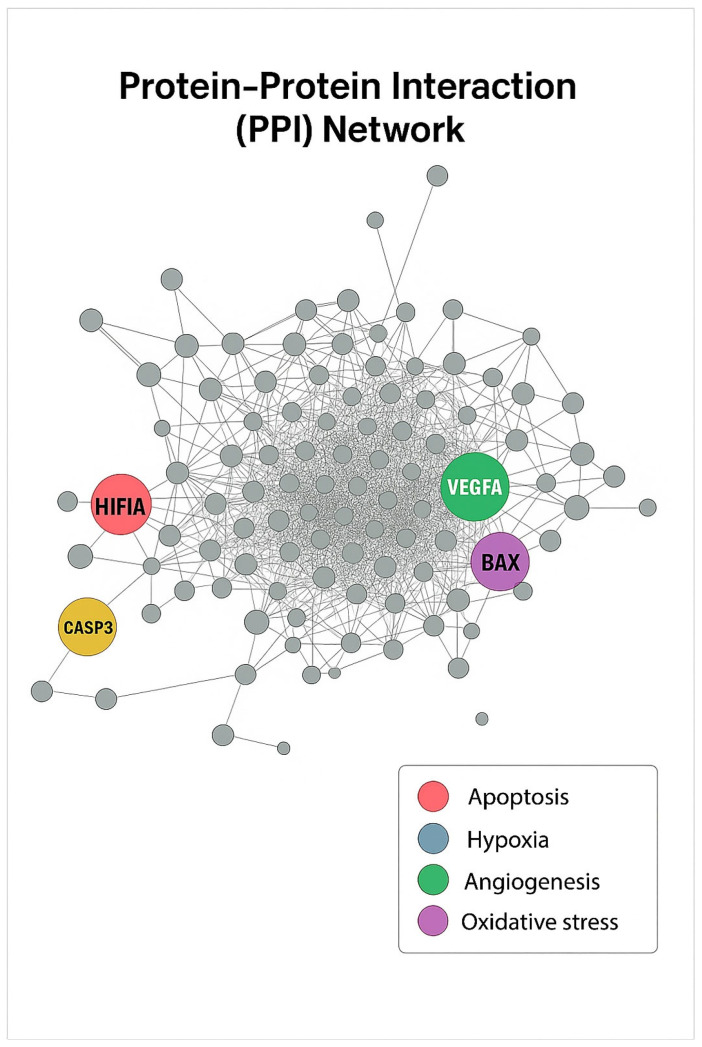
PPI Network. The PPI network was constructed using STRING (v12) and visualized in Cytoscape (v3.10). Each node represents a differentially expressed gene, and edges indicate protein–protein associations. Hub genes with high connectivity were identified as HIF1α (blue, hypoxia), CASP3 (red, apoptosis), VEGFA (green, angiogenesis), and BAX (purple, oxidative stress). These central nodes are proposed to mediate key molecular interactions underlying the combined antitumor effects of Q and Gem.

A PPI network was constructed using the differentially expressed genes identified in the combination treatment via the STRING database (v12) and visualized in Cytoscape (v3.10). The resulting network consisted of 326 nodes and 785 edges, representing functional associations among upregulated and downregulated proteins. Network topology analysis revealed that HIF1α, VEGFA, CASP3, and BAX occupied central hub positions, indicating their critical involvement in the regulatory network (Figure 12). These hub genes exhibited the highest degree of connectivity and betweenness centrality, suggesting that they serve as key molecular mediators linking apoptosis, hypoxia response, and angiogenesis pathways. Overall, the PPI analysis underscores that the synergistic anticancer effect of Q and Gem is likely mediated through network-level modulation of pivotal signaling nodes, particularly those integrating apoptotic and hypoxia-related mechanisms.

## 4. Discussion

Breast cancer progression is strongly influenced by the cellular redox balance, metabolic adaptation, and resistance mechanisms shaped within the hypoxic tumor microenvironment. In this study, we examined the combined cytotoxic and redox-modulating effects of quercetin, a flavonoid with known antioxidant and pro-apoptotic activity, and gemcitabine, an antimetabolite chemotherapeutic widely used in clinical oncology, on MDA-MB-231 triple-negative breast cancer cells. The findings of our study demonstrated that the combination of Q and Gem exhibited synergistic cytotoxic effects on MDA-MB-231 triple-negative breast cancer cells. These results are consistent with similar studies in the literature. A study by Liu et al. in 2020 reported that Q increased chemotherapeutic efficacy in Gemcitabine-resistant cancer cells by inducing apoptosis [11]. Similarly, in our study, the combination of Q and Gem resulted in significantly higher apoptosis rates compared to single treatments. In particular, the 76% decrease in viability in the Q100 + Gem10 combination can be considered a strong indicator of this synergistic effect. A comprehensive review by Li et al. published in 2017 highlights the potential of dietary natural products in the prevention and treatment of breast cancer and suggests that the combination of flavonoids such as Q with chemotherapy drugs may increase therapeutic efficacy [10]. The IC50 values and synergy indices in the combination groups obtained in our study support this hypothesis. In light of these findings in the literature, the mechanism by which Quercetin potentiates the antitumor activity of Gem may be through modulation of oxidative stress and activation of apoptosis signaling pathways.

The integration of biochemical assays, combination index (CI) analysis, and transcriptomic validation (GSE75168 dataset) demonstrated that the Q + Gem combination exerts a synergistic antitumor effect primarily through enhanced oxidative stress, mitochondrial apoptosis activation, and suppression of hypoxia-driven angiogenic signaling. Both compounds significantly reduced cell viability in a dose-dependent manner, while combination treatment produced a more potent cytotoxic response. A positive Bliss value and CI < 1 across multiple effect levels confirmed the synergistic interaction. These findings are consistent with previous studies reporting that flavonoids can enhance the chemosensitivity of cancer cells by modulating redox homeostasis and apoptotic pathways [14,15].

A prominent increase in intracellular ROS levels was observed following treatment. Q alone increased ROS production by ~1.6 fold, Gem by ~2.4 fold, and the combination by ~2.8-fold, indicating that co-treatment generates a stronger oxidative stimulus. Elevated ROS destabilizes mitochondrial integrity and promotes caspase activation [16]. Consistent with this mechanism, caspase-3 activity increased 2.1-fold (Q), 3.0-fold (Gem), and 3.6-fold (Q + Gem), demonstrating amplified mitochondrial apoptosis under combination treatment. Flow cytometry further supported these findings: total apoptotic cell rate increased from 2.8% (control) to 24.6% (Q), 38.4% (Gem), and 44.2% (Q + Gem). Additionally, intracellular ROS levels strongly correlated with apoptosis rate (r ≈ 0.9, *p* < 0.05), suggesting that oxidative stress is tightly linked to apoptotic cell death in MDA-MB-231 cells. Similar ROS-driven chemosensitization effects of flavonoids have been documented previously [17,18,19]. An important consideration arising from these findings is that synergistic cytotoxicity does not require a strictly synergistic increase in each individual upstream biological parameter. In the present study, although ROS generation and apoptosis-related readouts in the combination group were in some cases additive rather than synergistic, the overall loss of cell viability was clearly synergistic as confirmed by CI and Bliss analyses.

This apparent discrepancy can be explained by a convergence of stress mechanisms rather than amplification of a single pathway. Specifically, the combination treatment induced antioxidant exhaustion, as evidenced by disproportionate GSH depletion, together with sustained ROS exposure and marked suppression of HIF-1α/VEGF-mediated survival signaling. This coordinated disruption of redox homeostasis and adaptive survival responses provides a mechanistic explanation for the synergistic cytotoxicity observed. Importantly, the synergistic cytotoxicity observed in CI and Bliss analyses correlated most strongly with parameters reflecting redox collapse rather than with maximal increases in individual apoptotic markers. Among the evaluated endpoints, disproportionate depletion of intracellular GSH together with sustained ROS exposure and suppression of HIF-1α/VEGF-mediated survival signaling showed the closest temporal and mechanistic association with loss of cell viability. These findings indicate that the combination treatment drives cells beyond their adaptive redox threshold, resulting in irreversible stress and cell death, even when individual apoptotic readouts display predominantly additive changes.

At the molecular level, RT-qPCR analyses demonstrated that combination therapy significantly modulated key genes involved in apoptosis, hypoxia response, and angiogenesis. Bax and Caspase-3 were markedly upregulated, while anti-apoptotic Bcl-2 was downregulated, confirming mitochondrial apoptosis induction. HIF-1α and VEGF expression levels were reduced by approximately 80% and 60%, respectively, indicating that Q + Gem suppresses hypoxia-driven adaptive and angiogenic signaling. Previous findings showing that Q can inhibit HIF-1α/VEGF signaling and impair angiogenesis further support these observations [20,21]. Transcriptomic validation using the GSE75168 dataset confirmed consistent modulation of apoptosis-, hypoxia-, and angiogenesis-related pathways, strengthening the mechanistic interpretation of our in vitro results. HIF-1α is a master regulator of cellular adaptation to hypoxia, promoting VEGF-mediated angiogenesis and survival signaling in cancer cells. Downregulation of HIF-1α and VEGF by Q and Gem likely reduces these pro-survival pathways, thereby sensitizing MDA-MB-231 cells to apoptosis. This mechanism complements ROS-mediated oxidative stress, suggesting a dual pro-apoptotic effect of the treatments: direct induction of oxidative damage and inhibition of hypoxia-driven survival signals. The synergistic effect observed with the Q + Gem combination highlights the therapeutic potential of simultaneously targeting oxidative stress and HIF-1α/VEGF pathways. Hypoxia is known to play a critical role in the aggressive phenotype of triple-negative breast cancer. Srivastavag et al.’s (2023) recent study on the relationship between hypoxia and TNBC revealed that the hypoxic microenvironment is a significant factor in chemotherapy resistance [22]. In our study, the combination of Quercetin and Gemcitabine was observed to downregulate HIF-1α expression. This finding suggests that combination therapy may overcome hypoxia-induced chemotherapy resistance. HIF-1α, stabilized under hypoxic conditions, is known to contribute to cancer progression by regulating processes such as angiogenesis, cell migration, and metabolic adaptation. Inhibiting the HIF-1α signaling pathway in our combination therapy has the potential to control the aggressive behavior of TNBC. This mechanism may enhance treatment efficacy, particularly in the hypoxic core regions of solid tumors.

RNA-seq–based differential expression analysis revealed that combination treatment modulated nearly twice as many genes as single-agent treatments (853 DEGs), particularly in biological processes linked to apoptosis, hypoxia response, and angiogenesis. KEGG enrichment identified PI3K/Akt, MAPK, HIF-1, and VEGF signaling pathways as major targets, suggesting that the Q + Gem combination exerts a broad-spectrum antitumor effect by concurrently suppressing pro-survival and angiogenic pathways [9,23].

The PPI network analysis using the STRING database identified 326 nodes and 785 connections, with HIF1α, VEGFA, CASP3, and BAX identified as hub genes with high connectivity. The centrality of these genes in the network supports the critical role of apoptosis and hypoxia signaling networks in the anticancer effects of Q and GEM. Among the hub genes identified in the PPI network, HIF1A and VEGFA are central regulators of angiogenesis and cellular adaptation to hypoxia, whereas CASP3 and BAX are key mediators of apoptosis execution and mitochondrial membrane permeabilization. The connectivity of these genes underscores the coordinated regulation of mitochondrial apoptosis and hypoxia adaptation in the synergistic mechanism of Q + Gem. HIF1α and VEGFA were major nodes within angiogenesis and hypoxia-regulated networks, while CASP3 and BAX were key mediators of apoptosis execution, suggesting functional convergence of these pathways under combination treatment.

Our findings are supported by recent literature on antioxidants, which highlights the critical role of redox equilibrium in cancer cell survival. Brandl et al. reported that excessive ROS disrupts oncogenic redox homeostasis and induces apoptosis once buffering capacity is exceeded [24]. The enhanced oxidative stress and mitochondrial dysfunction observed in the Q + GEM group align with this model. Moreover, Gu et al. emphasized that adaptive antioxidant systems regulated through the KEAP1/NRF2 axis contribute to chemoresistance [25]. In our study, alterations in oxidative stress parameters (SOD, CAT, GSH, MDA) suggest that the combination treatment interferes with redox adaptation, thereby sensitizing cells to oxidative stress-mediated apoptosis [25].

**Figure 13:**
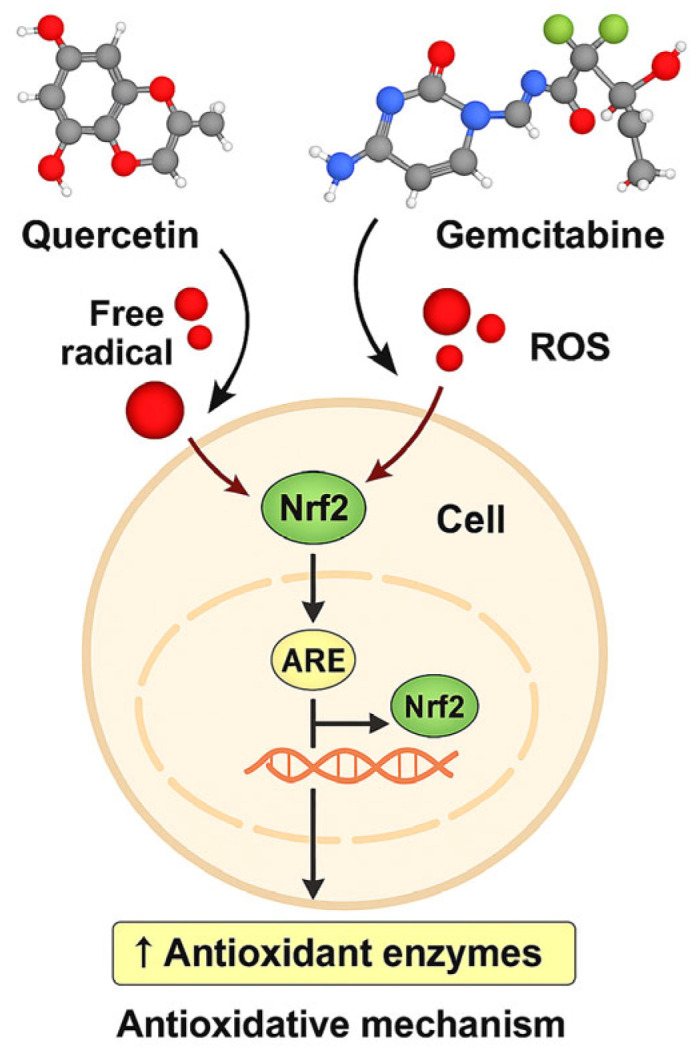
Schematic of the proposed Nrf2/ARE-centered redox mechanism of Q and GEM in MDA-MB-231 cells. Q modulates ROS and activates Nrf2/ARE, inducing antioxidant enzymes (SOD, CAT), while GEM increases ROS. Combined treatment overwhelms antioxidant defenses, causing GSH depletion, mitochondrial dysfunction, and apoptosis (↑Bax, ↑Caspase-3, ↓Bcl-2), alongside downregulation of HIF-1α/VEGF, suppressing survival and angiogenesis.

Taken together, these findings indicate that the co-treatment of Quercetin and Gemcitabine exerts a synergistic redox-modulating effect characterized by an initial adaptive antioxidant response followed by redox exhaustion under sustained oxidative stress. Quercetin acts as a potent free radical scavenger that enhances the nuclear translocation of Nrf2, thereby promoting the transcriptional activation of antioxidant response elements (ARE) and upregulation of downstream antioxidant enzymes such as SOD and CAT, and non-enzymatic antioxidant GSH. Meanwhile, Gemcitabine-induced oxidative stress and ROS accumulation serve as additional stimuli for Nrf2 activation, reinforcing the adaptive redox defense response. The proposed molecular mechanism underlying this interaction is summarized in Figure 13, which illustrates how the balance between ROS generation and antioxidant enzyme expression contributes to redox homeostasis and apoptosis regulation in MDA-MB-231 cells. Recent studies indicate that the Nrf2 signaling pathway plays a dual role in cancer cells. Lin et al. (2023) reported that while Nrf2 activation has a cytoprotective effect in the early stages, its prolonged activation can trigger pro-apoptotic signals [26]. The high ROS levels and increased apoptosis observed in our study support this second mechanism.

In line with the recent report by Liu et al., which emphasized both the therapeutic potential and challenges associated with targeting redox balance in cancer therapy, our findings provide experimental evidence that natural flavonoids such as Q can function as effective redox modulators to enhance chemotherapeutic response. Rather than acting as simple ROS scavengers, flavonoids appear to fine-tune intracellular redox dynamics, thereby sensitizing cancer cells to oxidative stress-induced apoptosis. Collectively, these insights support the growing concept that strategic modulation of redox signaling represents a promising approach for overcoming chemoresistance in triple-negative breast cancer [27].

Although this study provides comprehensive evidence for the synergistic cytotoxic and redox-modulating effects of Q and GEM in MDA-MB-231 triple-negative breast cancer cells, several limitations should be acknowledged. First, all experiments were conducted in vitro using a single MDA-MB-231 triple-negative breast cancer cell line; therefore, the results cannot fully reproduce the heterogeneity of MDA-MB-231 triple-negative breast cancer tumors observed in clinical settings. Second, the lack of in vivo analysis prevents assessment of pharmacokinetics, tissue distribution, and potential systemic toxicity—all of which are necessary to confirm the translational relevance of the observed synergistic effects. Third, while transcript-level evaluations revealed key mechanistic targets, protein-level validation was not performed; future studies should incorporate these techniques to strengthen mechanistic conclusions. Additionally, the study did not evaluate combination effects in non-malignant breast epithelial cells, which would be essential to assess potential off-target toxicity and therapeutic selectivity. Finally, although the transcriptomic dataset (GSE75168) provided supportive in silico evidence, multi-omics approaches such as proteomics or metabolomics may further clarify the interplay between oxidative stress, metabolic adaptation, and apoptotic signaling.

Future studies should investigate the combined Q–GEM regimen using xenograft or orthotopic TNBC models to validate in vivo efficacy, safety, and biodistribution. Expanding the analysis to additional breast cancer subtypes and patient-derived organoids will help determine whether the observed synergistic interactions are specific to the MDA-MB-231 phenotype or represent a broader therapeutic effect. Moreover, deeper mechanistic exploration of Nrf2/ARE, PI3K/Akt, and HIF-1α/VEGF pathways at the protein and post-translational levels may further elucidate how combined redox stress and apoptotic signaling contribute to chemosensitization. Integrating computational modeling, multi-omics analyses, and clinical datasets could ultimately support the development of personalized redox-targeted therapeutic strategies to overcome chemoresistance in aggressive breast cancer subtypes.

## 5. Conclusions

In conclusion, our findings demonstrate that Q potentiates the antitumor activity of GEM in MDA-MB-231 triple-negative breast cancer cells through redox-dependent mechanisms. The combination treatment amplified oxidative stress and ROS generation, triggered mitochondrial-mediated apoptosis, and suppressed hypoxia-induced angiogenic signaling via HIF-1α and VEGF downregulation. Transcriptomic and PPI network analyses highlighted HIF1A, VEGFA, CASP3, and BAX as hub genes linking apoptosis, oxidative stress, and hypoxia pathways. These data collectively indicate that dual modulation of redox homeostasis and hypoxic adaptation underlies the synergistic cytotoxicity of the Q–GEM combination. This redox-based therapeutic approach offers a promising strategy to overcome chemoresistance in aggressive breast cancers, warranting further in vivo and translational studies.

To the best of our knowledge, this is one of the first studies integrating biochemical, molecular, and transcriptomic analyses to elucidate the redox-dependent synergistic mechanism of Q and GEM in triple-negative breast cancer cells.

## Data Availability

Publicly available transcriptomic data used for in silico analyses are available in the GEO repository (accession number: GSE75168). Experimental datasets generated in this study are available from the corresponding author upon reasonable request.

## Abbreviations

Q	Quercetin
GEM	Gemcitabine
Nrf2/ARE	Nuclear factor erythroid 2-related factor 2/Antioxidant Response Element
SOD	Superoxide dismutase
CAT	Catalase
GSH	Glutathione
MDA	Malondialdehyde
HIF-1α	Hypoxia-Inducible Factor 1-alpha
VEGF	Vascular Endothelial Growth Factor
ROS	Reactive oxygen species
CI	Combination Index
MTT	3-(4,5-Dimethylthiazol-2-yl)-2,5-diphenyltetrazolium bromide
DCFDA	2′,7′-dichlorofluorescin diacetate
PI	Propidium Iodide
pNA	p-nitroaniline
DGEA	Differential gene expression analysis
GO	Gene Ontology
KEGG	Kyoto Encyclopedia of Genes and Genomes
PPI	Protein–protein interaction
